# The relationship between work–family conflict and job satisfaction for preschool teachers in rural China: a moderated mediation model

**DOI:** 10.3389/fpubh.2023.1236713

**Published:** 2023-12-06

**Authors:** Yingjie Wang, Qianqian Xia, Huilan Yue, Ruiwei Yu, Wengui Zhang, Jing Li, Dan Chen, Pin Xu

**Affiliations:** ^1^School of Teacher Education, Huzhou University, Huzhou, China; ^2^Teaching and Research Office, Shanghai Hongkou Institute of Education, Shanghai, China

**Keywords:** work–family conflict, job satisfaction, occupational identity, social support, preschool teachers

## Abstract

**Background:**

Job satisfaction for preschool teachers in rural areas has an important impact on their professional development, physical and mental health, and the development of preschool education. However, few studies have explored the factors that influence rural preschool teachers’ job satisfaction.

**Purpose:**

This study aims to examine the influence of rural preschool teachers’ work–family conflict on their job satisfaction, and the mediating effect of occupational identity, the moderating effect of social support.

**Method:**

Participants included 3,065 rural preschool teachers from Zhejiang Province in mainland China. Teachers completed questionnaires on work–family conflict, occupational identity, job satisfaction, and social support. The correlation and moderated mediation analyses were conducted using SPSS PROCESS.

**Results:**

(1) work–family conflict is associated with poorer job satisfaction in preschool teachers; (2) occupational identity mediates the relationship between work–family conflict and job satisfaction; and (3) a high level of social support alleviates the negative influence of work–family conflict on job satisfaction and promotes the positive effect of occupational identity on job satisfaction.

**Conclusion:**

The study revealed the negative impact of work–family conflict on preschool teachers’ job satisfaction, and the protecting effect of social support, which has important implications for improving teachers’ future job satisfaction.

## 1 Introduction

With the development of preschool education in rural areas of China, the job satisfaction of preschool teachers in these areas has become a topic of interest. Job satisfaction refers to employees’ positive emotional reactions after evaluating their work or work experience, and also is an important indicator used to evaluate an individual’s job achievement and work value (1, 2). Teachers’ job satisfaction refers to the state in which teachers express their satisfaction and positive feelings toward their own work (3), which not only affects teachers’ working state and professional development, but also affects teaching effectiveness, the stability of the teaching staff, and children’s development (4, 5). Teachers with high levels of job satisfaction are more committed to their work and have higher work efficiency, which leads to rapid professional growth and improves the quality of early education (6). In addition, students have better learning experiences and are more engaged in learning when teachers have a high level of job satisfaction (7–9). On the contrary, teachers with low levels of job satisfaction are more susceptible to job burnout, low levels of engagement and occupational commitment, and higher levels of turnover intention (10, 11).

In China, job satisfaction for rural preschool teachers is low, which leads to high levels of job turnover intention in these areas (12, 13). Therefore, it is critical to explore the factors influencing preschool teachers’ job satisfaction and implement effective intervention to improve the quality of rural preschool education. According to the Conservation of Resources Theory, individuals have limited resources during certain periods of time (14), so when the resources of one domain are insufficient, the resources of another domain are consumed, resulting in a conflict between the two domains (15). Preschool teachers tend to be female, so they often shoulder the responsibilities of both family and work, which can lead to greater conflicts between work and family (16–18). When individuals cannot properly cope with conflicts between work and family, they experience negative emotions to protect their only resources and reduce their commitment to work (19–21). Therefore, it is of great significance to explore the influence of work–family conflict on the job satisfaction of rural preschool teachers.

### 1.1 Work–family conflict and job satisfaction

The Work-Family Interface Model indicates that when work and family responsibilities interfere with each other, individuals will experience a high level of work–family conflict (22). Work–family conflict often occurs when individuals face different role tasks, such as people have to face incompatible role pressure from work and family (23). Role conflict between family and work was divided into two dimensions. One is where work intrudes on family, in which too much of an individual’s time and energy are devoted to work, so they do not fulfill the demands of family roles and functions well. The other dimension is where family intrudes on work, which means an excessive burden from family will affect work engagement and the completion of work tasks (24, 25). Some empirical studies found that high level work–family conflict among preschool teachers had many negative effects on their work outcomes and physical and mental health, such as higher job stress, job burnout, and less satisfaction with their families (26, 27).

Rural preschool teachers are an unique group, who are on the front lines of rural education, have worse welfare benefits, poor working conditions and insufficient promotion opportunities than urban teachers (28), so they may face greater pressure from work, family, and life (29, 30). From this perspective, it is of great value to explore the work–family conflict of rural preschool teachers. Higher degree of work–family conflict will affect their physical and mental health and professional development (17, 31, 32). Previous studies on university professors, bank executives, and nurses have shown that work–family conflict negatively predicted job satisfaction (33–35). For example, Atteh et al. (36) found that conflict between work and family led to a decrease in employees’ work performance and reduced their enthusiasm and expectations for work. Khokhar et al. (37) found a significant negative relationship between work–family conflict and employees’ basic salary and welfare benefits. Among existing studies, few have focused on work–family conflict and job satisfaction in rural preschool teachers. Improving job satisfaction for rural preschool teachers is of great significance for the development of preschool education and child development. The current study aims to explore the influence of work–family conflict on job satisfaction in rural preschool teachers. Based on existing studies and a relevant theoretical basis, our first hypothesis is that work–family conflict among preschool teachers will lead to lower job satisfaction.

### 1.2 Occupational identity as a mediating role

Teacher’s occupational identity refers to the self-acceptance and recognition to their career in teaching and education based on their own practical experience and personal background, which includes the recognition degree of occupational demand, occupational cognition, occupational will and occupational emotion etc. (38), which is the result of an interaction between their personal experiences and the social, cultural, and institutional environment (39). Only by establishing an internal occupational identity, where teachers feel the happiness and life value brought about by their profession, can they truly realize their professional potential (40, 41). Role theory proposes that people play different roles in work and family. When individuals fail to meet the requirements of their roles during role conversion, they enter into a state of confusion, resulting in psychological distress during work, which in turn has a negative impact on work outcomes, such as occupational identity (42, 43). When teachers are unable to deal with the relationship between work and family effectively, they experience negative cognitions and emotions toward their careers, which reduces their occupational identity (18, 44, 45). In addition, according to the Spillover Hypothesis Theory, too much burden from work or family responsibilities will have spillover effects into other areas of an individual’s life. Work–family conflict is a type of negative spillover (46). The negative emotional attitude generated by work–family conflict will extend to work and individuals may suffer from greater pressure or job burnout, which affects their views and attitudes toward their careers (47).

Empirical studies also show that work–family conflict can significantly reduce young people’s occupational identity (48). When teachers’ work and family interact and become one of the main stressors of their career development, this can result in a negative attitude toward their career, and a decrease in the level of occupational identity (44, 49). One study found that nurses with a high level of work–family conflict also experienced long-term emotional exhaustion, which had a serious negative impact on their occupational identity (50). Studies also have suggested that occupational identity significantly impacts job satisfaction (51, 52). According to social identity theory, the level of occupational identity affects one’s attitude toward work and leads to a higher level of job satisfaction (53). Occupational identity is one of the internal motivating factors that can connect teachers’ personal values with their professional values, resulting in higher job satisfaction (54). In conclusion, based on theoretical and empirical research, our second hypothesis is that the occupational identity of rural preschool teachers will mediate the relationship between work–family conflict and job satisfaction.

### 1.3 Social support as a moderating role

According to the buffer theory of social support, positive social support can reduce the negative effect on individuals in the face of adverse situations (55). Social support refers to the resources available to individuals when needed, which encompasses expressive or instrumental provisions (perceived or received) provided by the community, social networks, and trustworthy individuals, applicable in both everyday scenarios and crises (56–58). Good social support can make individuals feel the support from society, family, friends, organizations, etc., which can increase positive self-perception, improve their mental health, and provide emotional support (59). Researchers have found that social support has a positive effect on individuals’ career development by reducing role pressure at work and increasing job satisfaction and well-being (60, 61). Social support can increase employees’ positive feelings about family life and work, help them improve balance between work and family, and improve job satisfaction (62). Some evidence also shows that social support is a moderating factor of the negative outcomes caused by stress (63, 64), with support from the workplace, family, and friends moderating the negative correlation between high levels of work–family conflict and job satisfaction (65). Based on these studies, our third hypothesis, that social support may play a moderating role in the relationship between preschool teachers’ work–family conflict and job satisfaction.

The value of social support in one’s personal as well as professional environment has been demonstrated, especially in buffering job burnout (66). People with high levels of social support can generate more psychological resources to cope with various pressures and conflicts, are more likely to maintain a healthy physical and mental state, and experience less job burnout (67–69). Some studies have shown that support from leaders and colleagues can reduce the negative effects of work–family conflict, such as job burnout, depression, poor physical health, and low work efficiency (68, 70). Glaveli et al. (71) also found that if companies provide more organizational support for employees, they will experience fewer work–family conflicts and higher job satisfaction. Employees with high levels of social support experience less work–family conflict and are better able to fulfill their responsibilities at home and work (72). Preschool teachers have heavy workloads and they also have daily responsibilities for taking care of their families. Owing to inadequate economic development and limited income for teachers in rural areas, the effort-reward imbalance may exacerbate their job burnout, then hinder their professional development. Moreover, lack of enough social support from organization (e.g., lack of professional guidance at work, lack of cooperation among colleagues) and family (e.g., family members’ support to their work) may further exacerbate difficulties in career development for preschool teachers. When pressures from work and family life become increasingly intense and teachers are unable to address them, it can easily lead to job burnout and a decrease in occupational identity (41). Therefore, social support may also be a buffer against the negative impact of work–family conflict on teachers’ occupational identity. Based on this, we propose our fourth hypothesis; social support may play a moderating role in the relationship between work–family conflict and preschool teachers’ occupational identity.

For rural preschool teachers, they may usually face various work demands, pressure from both work and family, which may make them have doubts about their occupation. And then for the teachers with low level occupational identity may also results in the decrease of job satisfaction, so social support are even more important for their physical and mental health and job performance (73). High level of social support from friends, colleagues, or family, will make their daily work easier, so that their identification with their occupation will be easier to translate into higher job satisfaction. However, teacher’s lack of social support may make their work more difficult and complex, and more work tasks and stress, which may restrain the positive effect of occupational identity on their job satisfaction. Study on beginning kindergarten teachers have shown that more social support can enhance the relationship between occupational identity and their professional well-being (74). So, based on the empirical studies, we propose a fifth hypothesis where social support plays a moderating role in the relationship between preschool teachers’ occupational identity and job satisfaction.

### 1.4 The present study

**Figure 1:**
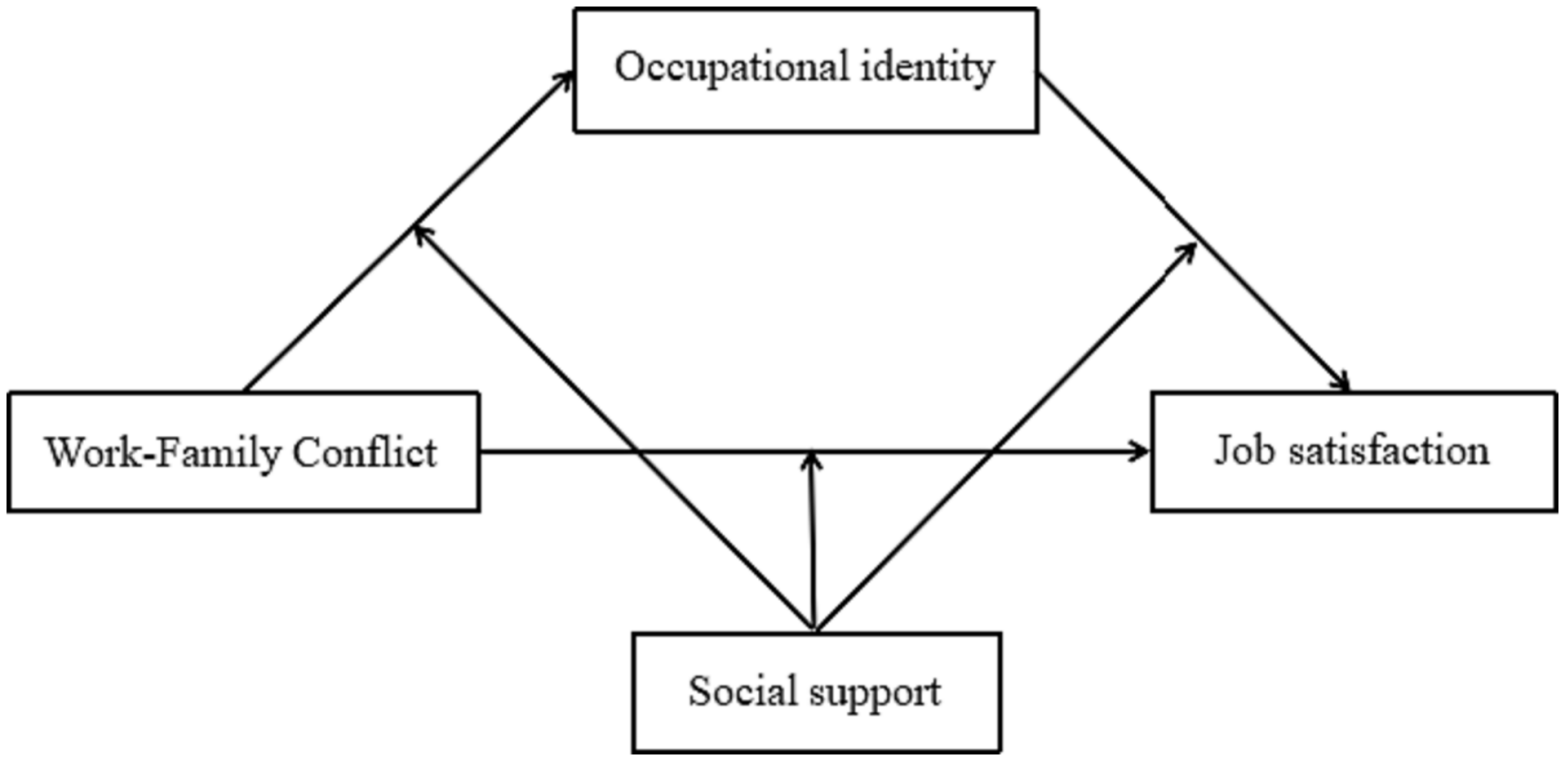
The proposed moderated mediation model.

To summarize, according to relevant theories and empirical studies, preschool teachers’ job satisfaction may be affected by factors such as work–family conflict, occupational identity, and social support, but there is no research exploring the relationship among these variables for rural preschool teachers. Therefore, this study aims to investigate the effects of work–family conflict on job satisfaction, and explore the mediating effect of occupational identity and the moderating effect of social support on job satisfaction among preschool teachers in a rural region of China. The proposed hypothetical model is illustrated in Figure 1.

## 2 Methods

### 2.1 Participants

In this study, 3,366 kindergarten teachers (for children aged 3–6 years old) from Zhejiang Province were recruited in November, 2022, after a preliminary check of the data, 301 of the questionnaires were excluded due to the regular answers (e.g., some teachers fill in the same number for all questions) or too much data was missing, then 3,065 rural kindergarten teachers were included. Among the participants, there were 3,030 females and 35 males, all from public kindergartens, ranging in age from 19 to 64 years old (*Mean*_age_ = 32.08 years, *SD* = 7.10 years). Most had less than 10 years teaching experience (32.95% 5 years or less; 27.22% 6–10 years; 18.24% 11–15 years; 9.53% 16–20 years; and 12.06% more than 21 years), and most had at least a bachelor’s degree (1.31% high school; 33.37% junior college; and 65.32% bachelor degree).

### 2.2 Measures

#### 2.2.1 Work–family conflict

Teachers’ work–family conflict was measured using the Chinese version of Work–Family Conflict Scale (WFCS) (75, 76). The WFCS consists of 10 items on two subscales: (1) work leads to family conflict (five items; e.g., “The demands of my job are affecting my family life”) and family leads to work conflict (five items; e.g., “My family life affects my work”). Participants rated each item on a five-point Likert scale (1 = completely disagree; 5 = completely agree). Higher scores indicated higher levels of work–family conflict. The scale shows good psychometric properties in the context of Chinese culture (75), and the Cronbach’α coefficient of the overall scale in this study was 0.91.

#### 2.2.2 Occupational identity

Teachers’ occupational identity was measured using the Occupational Identity Scale [OIS; (77)], which was developed by Wei and his colleagues in Chinese context. The OIS consists of 18 items that assess teachers’ occupational identity on four subscales: (1) role values (six items, e.g., “As a preschool teacher, I often feel respected”); (2) professional behavior tendency (five items, e.g., “I take my work seriously”); (3) professional values (four items, e.g., “I think my work plays an important role in the development of society); and (4) professional sense of belonging (four items, e.g., “I care about how others think of preschool teachers’ work”). Participants rated each item on a five-point Likert scale (1 = very inconsistent; 5 = very consistent), with a high score indicating a high level of occupational identity. The Cronbach’α coefficient for the OIS scale was 0.94.

#### 2.2.3 Job satisfaction

Teachers’ job satisfaction was measured using the Chinese version of Job Satisfaction Scale [JSS; (78, 79)]. The JSS consists of six items (e.g., “How satisfied are you with your job?”) assessing teachers’ job satisfaction. Participants rated each item on a five-point Likert scale (1 = very dissatisfied; 5 = very satisfied), with a high score indicating a high level of teachers’ job satisfaction. The scale has shown good psychometric properties in Chinese context (79). The Cronbach’α coefficient for the JSS in this study was 0.94.

#### 2.2.4 Social support

Teachers’ social support was measured using the Chinese version of Perceived Social Support Scale [PSSS; (80, 81)], PSSS was revised. The PSSS consists of 12 items assessing teachers’ perceived level of social support using three subscales: (1) support from family (four items, e.g., “My family is willing to help me make decisions”); (2) support from friends (four items, e.g., “When I face difficulties, I can rely on my friends”); and (3) other support (four items, e.g., “When I have a problem, some people will be there for me”). Participants rated each item on a seven-point Likert scale (1 = completely disagree; 7 = completely agree), with a high score indicating a high level of support from others. The scale has previously shown satisfactory psychometric properties in Chinese context (80). The Cronbach’α coefficient for the PSSS was 0.96.

#### 2.2.5 Control variables

For our study, we also collected demographic variables (e.g., teachers’ gender, age, teaching experience, educational background, and income) as control variables, which have been demonstrated to be related to the outcome variables (82, 83). Therefore, these demographic variables were included as control variables in the mediation and moderation analyses.

### 2.3 Procedure

All participants were recruited from kindergartens in Zhejiang Province in China using an online survey. Before collecting the data, the study received strict ethical approval from the researcher’s university. We contacted the heads of preschool education in the 21 rural areas in Zhejiang Province, and they helped us to select several kindergartens in each rural area randomly, and contacted the kindergarten principals. We sent the link of the online survey to the principals, who forwarded it to the each teacher. The first page of the online survey is the informed consent, all participants were informed of the purpose and details of the study and signed the informed consent. If the teachers agree to participate in the survey, they can click “agree” to go to the page of survey, if they do not agree to participate in the survey, they can click “disagree” to quit directly.

### 2.4 Analytic strategy

We used SPSS 22.0 to calculate the correlations, descriptive statistics, and Cronbach’α coefficients for each variable in this study. Mediation and moderated mediation analysis were conducted using SPSS PROCESS (84). We used a bootstrapping procedure with 5,000 resamples to assess the unconditional indirect effects, which were considered significant when the 95% bias-corrected and accelerated confidence intervals (95% CI) did not contain zero.

## 3 Results

### 3.1 Preliminary analyses

The means, standard deviations, and correlations for work–family conflict, occupational identity, job satisfaction, social support, and demographic variables are displayed in Table 1. The results indicated that teachers’ demographic variables (gender, age, teaching experience, educational background, and income) were significantly correlated with study outcomes. The main study variables were significantly correlated each other.

**Table 1:** Correlations and descriptive statistics for study variables.

	1	2	3	4	5	6	7	8	9
1. Gender	-								
2. Age	0.02	-							
3. Teaching experience	0.02	0.84^***^	-						
4. Educational background	0.02	0.06^*^	0.10^***^	-					
5. Income	−0.05^*^	0.09^***^	0.16^***^	0.33^***^	-				
6. Work–family conflict	−0.01	−0.07^***^	−0.05^*^	0.04^*^	0.02	-			
7. Occupational identity	0.01	0.06^**^	0.06^**^	0.06^***^	0.08^***^	−0.28^***^	-		
8. Job satisfaction	−0.03	0.01	−0.01	−0.01	0.08^***^	−0.45^***^	0.40^***^	-	
9. Social support	0.01	0.06^***^	0.06^**^	0.03	0.03	−0.39^***^	0.47^***^	0.45^***^	-
*M*	-	32.08	9.66	-	-	2.43	4.32	3.68	5.66
*SD*	-	7.10	7.23	-	-	0.78	0.57	0.83	1.10

^*^p < 0.05, ^**^p < 0.01, and ^***^p < 0.001; gender: 0 = male, 1 = female; education background: 1 = high school, 2 = junior college, and 3 = bachelor’s degree; income: 1 = under 80,000 RMB per year, 2 = 80,000–100,000 RMB per year, 3 = 100,000–120,000 RMB per year, and 4 = above 120,000 RMB per year.

To examine the influence of the categorical demographic variables (gender, educational background, and income) on the outcome variables (occupational identity and job satisfaction), we conducted independent sample T-test and ANOVAs. The results showed that, there is no significant gender difference on teachers’ occupational identity (*t* = 1.79, *p* > 0.05) and job satisfaction (*t* = −0.73, *p* > 0.05). There is significant educational background difference on occupational identity (*F* = 7.10, *p* < 0.01), but no significant difference on job satisfaction (*F* = 0.29, *p* > 0.05). The *post hoc* test results showed that, teachers with bachelor’s degree have higher occupational identity than teachers with high school and junior college. There is significant income difference on occupational identity (*F* = 6.61, *p* < 0.001) and job satisfaction (*F* = 8.35, *p* < 0.001). The *post hoc* test results showed that, teachers with the income of “under 80,000 RMB” have lower occupational identity and job satisfaction than the other three categories of income, teachers with the income of “80,000–100,000 RMB” have lower occupational identity and job satisfaction than the other two higher income categories.

### 3.2 The mediation analysis

**Figure 2:**
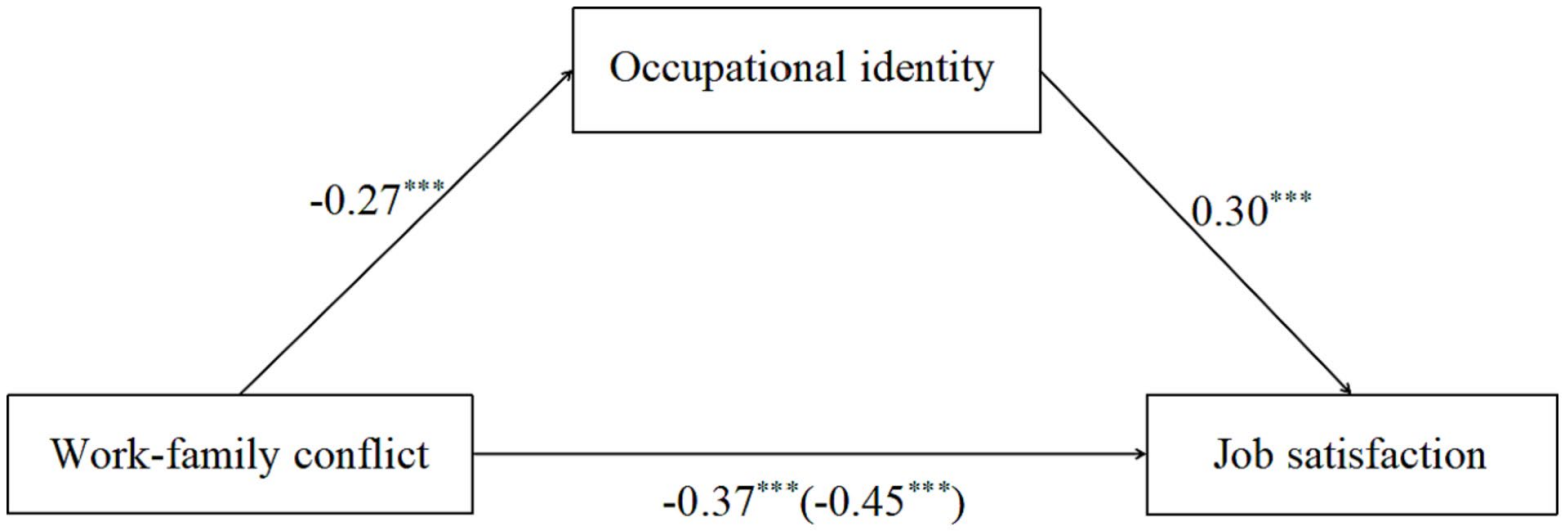
The mediating effect of occupational identity. ^***^p < 0.001.

A mediation analysis was conducted with model 4 in SPSS PROCESS to examine the mediating effect of occupational identity between teachers’ work–family conflict and job satisfaction. Because the teachers’ demographic information correlated with the main study variables, we also included these variables (teachers’ gender, age, teaching experience, educational background, and income) as control variables. All the study variables were standardized before the mediation analysis. The results (displayed in Figure 2 and Table 2) showed that, work–family conflict had a significant negative effect on job satisfaction (*β* = −0.45, *p* < 0.001), after adding occupational identity in the model, work–family conflict also negatively predicted job satisfaction (*β* = −0.37, *p* < 0.001). Work–family conflict also significantly predicted occupational identity (*β* = −0.27, *p* < 0.001), and occupational identity positively predicted job satisfaction (*β* = 0.30, *p* < 0.001). The bias-corrected bootstrap method was used to test the mediating effect. There were 5,000 bootstrap samples ran by PROCESS, which indicated that the indirect effect was −0.08, 95%*CI* = [−0.10, −0.07].

**Table 2:** Results of the mediation model.

Dependent variables	Independent variables	*R*	*R^2^*	*F*	*β*	*t*	*95%CI*
Job satisfaction		0.46	0.21	130.19^***^			
	Gender				−0.26	−1.71	[−0.57, 0.04]
	Age				0.01	1.14	[−0.01, 0.01]
	Teaching experience				−0.01	−2.19^*^	[−0.02, −0.01]
	Educational background				−0.05	−1.51	[−0.05, 0.08]
	Income				0.11	5.96^***^	[0.08, 0.15]
	Work–family conflict				−0.45	−27.12^***^	[−0.48, −0.42]
Occupational identity		0.29	0.08	44.84^***^			
	Gender				0.13	0.78	[−0.20, 0.45]
	Age				0.01	0.94	[−0.01, 0.01]
	Teaching experience				0.01	0.19	[−0.01, 0.01]
	Educational background				0.10	2.61^**^	[0.02, 0.17]
	Income				0.07	3.27^**^	[0.03, 0.11]
	Work–family conflict				−0.27	−15.30^***^	[−0.31, −0.24]
Job satisfaction		0.54	0.29	173.00^***^			
	Gender				−0.30	−2.07^*^	[−0.59, −0.02]
	Age				0.01	0.88	[−0.01, 0.01]
	Teaching experience				−0.01	−2.38^*^	[−0.02, −0.01]
	Educational background				−0.08	−2.48^*^	[−0.14, −0.02]
	Income				0.09	5.18^***^	[0.06, 0.13]
	Occupational identity				0.30	18.43^***^	[0.28, 0.33]
	Work–family conflict				−0.37	−22.55^***^	[−0.40, −0.33]

^*^p < 0.05, ^**^p < 0.01, and ^***^p < 0.001.

### 3.3 The moderated mediation analysis

After testing the mediating effect of occupational identity, we tested the moderating effect of social support in the mediation model using Model 59 from SPSS PROCESS. The 95%*CI* does not contain zero means the moderated mediation effects are significant. The results (see Table 3) showed that social support had a moderating effect on the direct effect of work–family conflict on job satisfaction (*β* = −0.04, *t* = 2.63, *p* < 0.01, 95%*CI* [−0.07, −0.01]), and it also moderated the relationship between occupational identity and job satisfaction (*β* = 0.03, *t* = 2.73, *p* < 0.01, 95%*CI* [0.01, 0.06]), but the moderated effect of work–family conflict on occupational identity was not statistically significant (*β* = −0.02, *t* = −1.40, *p* > 0.05, 95%*CI* [−0.05, 0.01]). So, the results showed that the moderated mediation effect was significant, teachers’ social support moderated the relationship between work–family conflict and job satisfaction, occupational identity and job satisfaction. The indirect effects of work–family conflict on job satisfaction via occupational identity at high level of social support (*β* = −0.03, *SE* = 0.01, 95%*CI* [−0.05, −0.02]) and low level social support (*β* = −0.02, *SE* = 0.01, 95%*CI* [−0.03, 0.001]).

**Table 3:** Results of the moderated mediation model.

Dependent variables	Independent variables	*R*	*R^2^*	*F*	*β*	*t*	*95%CI*
Occupational identity		0.48	0.23	112.36^***^			
	Gender				0.13	0.89	[−0.16, 0.43]
	Age				0.01	0.43	[−0.01, 0.01]
	Teaching experience				0.01	0.30	[−0.01, 0.01]
	Educational background				0.06	1.83	[−0.01, 0.13]
	Income				0.06	3.17^**^	[0.02, 0.10]
	Work–family conflict				−0.11	−5.98^***^	[−0.14, −0.07]
	Social support				0.42	24.01^***^	[0.39, 0.46]
	Work–family conflict × Social support				−0.02	−1.40	[−0.05, 0.01]
Job satisfaction		0.58	0.34	148.98^***^			
	Gender				−0.31	−2.16^*^	[−0.58, −0.03]
	Age				0.01	0.70	[−0.01, 0.01]
	Teaching experience				−0.01	−2.40^*^	[−0.02, −0.01]
	Educational background				−0.10	−2.99^**^	[−0.16, −0.03]
	Income				0.09	5.40^***^	[0.06, 0.13]
	Work–family conflict				−0.29	−17.30^***^	[−0.32, −0.26]
	Occupational identity				0.22	12.18^***^	[0.18, 0.25]
	Social support				0.25	13.91^***^	[0.22, 0.29]
	Work–family conflict × Social support				−0.04	−2.63^**^	[−0.07, −0.01]
	Occupational identity × Social support				0.03	2.73^**^	[0.01, 0.06]

^*^p < 0.05, ^**^p < 0.01, and ^***^p < 0.001.

**Figure 3:**
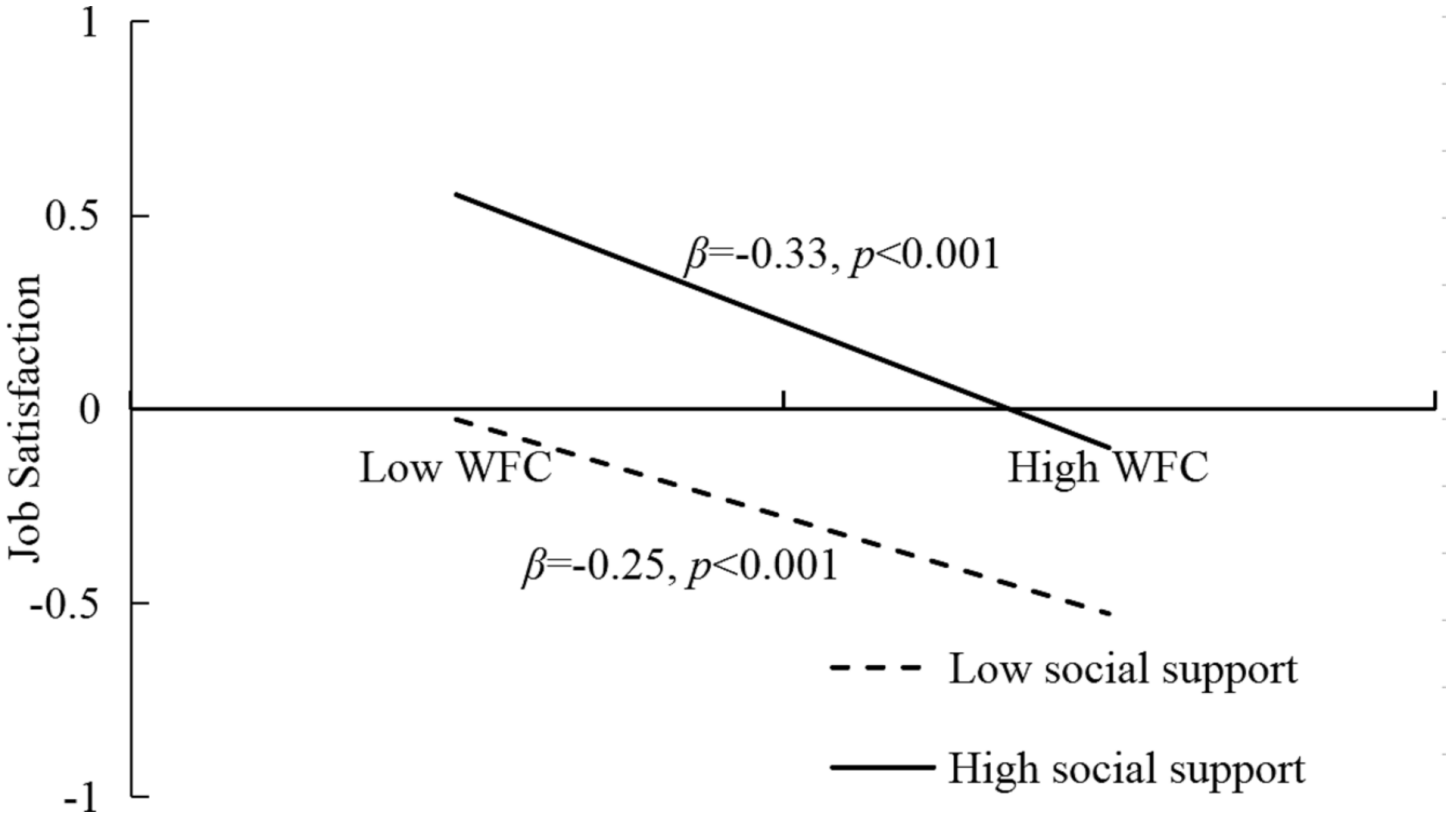
The moderating effect of social support between work–family conflict and job satisfaction. WFC, Work–family conflict.

**Figure 4:**
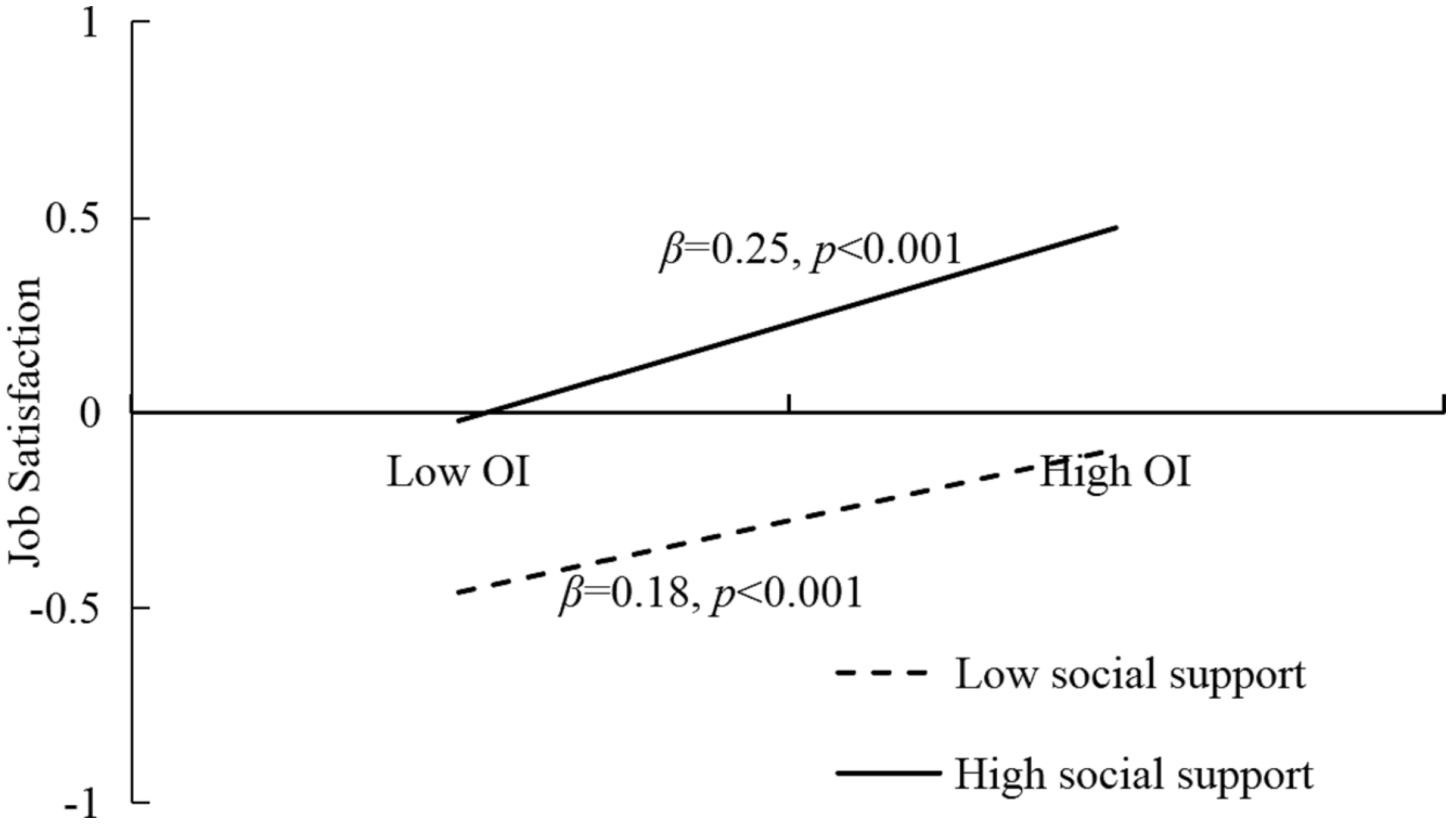
The moderating effect of social support between occupational identity and job satisfaction. OI, Occupational identity.

In order to reveal the essence of the interaction, then we conducted the simple slopes at lower (−1SD) and higher (+1SD) levels of social support (see Figures 3, 4), the predictive effect of work–family conflict on job satisfaction at high level of social support (*β* = −0.33, *t* = −15.29, *p* < 0.001) was higher than a low level of social support (*β* = −0.22, *t* = −11.04, *p* < 0.001). Further, for the moderating effect of social support on the relationship between occupational identity and job satisfaction, the predictive effect of occupational identity on job satisfaction at high level of social support (*β* = 0.25, *t* = 10.59, *p* < 0.001) was higher than a low level of social support (*β* = 0.18, *t* = 9.73, *p* < 0.001).

### 3.4 Robustness test

In this study, some data were excluded because of the “regular answers.” In order to enhance the reliability of the results, a robustness test was conducted. We used the data includes the “regular answers” to perform another set of analysis. The results of the moderated mediation model showed that there were no differences between the two sets of analyses (one with the regular answers and one without). However, in order to make the study more rigorous and scientific, we excluded the “regular answers” during the actual analysis.

## 4 Discussion

### 4.1 The relationships between work–family conflict and job satisfaction

The current study findings showed that the work–family conflict of rural preschool teachers had a negative predictive effect on their job satisfaction, which was consistent with previous research (17). According to Boundary Theory, individuals will establish different role boundaries around their work field or family field, and they devote themselves to work and family domain and carry out cross-boundary role change activities every day (85). Rural preschool teachers who experience work–family conflict do not have enough time and energy to devote themselves to their work, depleting their work resources, affecting their work performance, and leading to a potential decrease in job satisfaction (17, 86). Influenced by traditional Chinese culture, family is highly valued, especially among women in rural areas (87). Most preschool teachers in rural areas are female, so they not only need to fulfill the expectations of a female’s family role, but also shoulder work responsibilities (29). When work interferes with family, they are more likely to attribute work–family conflict to work and be dissatisfied with their work (18).

### 4.2 The mediating role of occupational identity

The current study found that the occupational identity of rural preschool teachers played a mediating role between work–family conflict and job satisfaction. Previous studies found that work–family conflict could affect preschool teachers’ negative work emotions and reduce their occupational identity, which is similar to this result (44, 48). The results also confirmed the Conservation of Resources Theory, where individuals strive to preserve resources (14). Work–family conflict consumes a lot of resources, leading to a loss of employees’ emotional resources, low organizational loyalty, and a decline in job satisfaction (88). When rural preschool teachers cannot cope with work–family conflicts well, these conflicts will reduce the consistency between their developmental goals and career development requirements, which can negatively affect their career self-evaluations and lead to decreases in occupational identity (49). However, individuals with high levels of occupational identity have a deep understanding of their work, are more motivated and enthusiastic about work, which increases their job satisfaction (41, 89).

### 4.3 The moderating effect of social support

First, in the current study, social support had a statistically significant moderating effect on the relationship between work–family conflict and job satisfaction for rural preschool teachers. In other words, social support could help alleviate the negative impact of work–family conflict on job satisfaction. This result is in line with the buffering theory of social support, which suggests that a high level of social support can help alleviate the adverse effects of physical and mental conditions under stressful conditions (55). Rural preschool teachers with more social support are able to receive more help from others to deal with difficulties and stressors, while those with less social support are prone to experience burnout (90), and high level of job burnout can reduce job satisfaction and work efficiency (91). According to the Conservation of Resources Theory, social support as a kind of positive psychological resource that can moderate this negative impact of work–family conflict and enhance job satisfaction (15, 65). Therefore, rural preschool teachers with more social support can regulate their physical and mental states more quickly, redevote themselves to work, and increase job satisfaction (18).

Second, this study found that social support moderated the association between occupational identity and job satisfaction. In other words, compared with rural preschool teachers with low levels of social support, those with more social support can maintain higher job satisfaction when they have a lower occupational identity (45). Previous studies have also found that social support promotes psychological and occupational traits, which can enhance individuals’ work attitudes, thus affecting their career cognition (60, 92). Only when individuals establish a strong internal sense of occupational identity can they be motivated and satisfied with their work (93). Because of low level of welfare and poor working conditions of rural preschool teachers, help and support from family members, colleagues, and friends are particularly important, which can help alleviate the negative impact of low occupational identity on rural preschool teachers’ job satisfaction.

Finally, this study did not find a moderating effect of social support on the relationship between work–family conflict and occupational identity. The main reason was that, when rural preschool teachers encounter work–family conflicts, especially when heavy work tasks interference with family life, the supports from the organization can help them solve practical problems, which may be particularly important for alleviating the negative impact on professional identity. However, in the measurement of social support in this study, the component of organizational support is little, which may lead to the moderating effect of social support being insignificant. Currently, there are few studies on this topic; thus, the possible underlying mechanisms need to be further explored. Therefore, we should pay more attention to the attitudes and emotions of rural preschool teachers toward their careers and encourage additional support and affirmation from family, friends, leaders, and society to alleviate the conflicts they face between work and family.

### 4.4 Implications and limitations

Through a survey of 3,605 rural preschool teachers in China, this study found a mediating effect of occupational identity between work–family conflict and job satisfaction, as well as a moderating effect of social support. These results have important theoretical and practical implications. First, the results of the moderated mediation model further expanded the interpretation scope of the Conservation of Resources Theory and the Buffering Model of Social Support. Second, the results revealed the current situation of rural preschool teachers’ work–family conflict, and highlighted the need to examine the resulting physical and mental health and professional development problems. Finally, the results showed that social support can alleviate many problems in teachers’ professional development and enhance their job satisfaction. Therefore, finding ways to provide more support to rural preschool teachers should be considered. For example, programs of social and emotional learning (SEL) for preschool teachers have been proved to promote their social and emotional competence, reduce their stress, and improve the quality of teacher-child interactions effectively (94). The SEL program is also one of the ways of social support to relieve the negative affect due to work–family conflict.

There were some limitations to this study. First, a cross-sectional design was used to collect data, which could not strictly explain the causal relationships among the variables. A longitudinal study design should be used in future studies. Second, the data in this study were self-report, which is prone to common method bias. Therefore, data should be collected in a multi-reporting manner in future studies. Third, considering that some teachers may be sensitive about the investigation, some relevant covariates (e.g., marital status and caregiving responsibilities) were not included in this study. In future studies, we will take appropriate ways to solve this problem, and consider the impact of these factors on the hypothetical model.

## Data availability statement

The raw data supporting the conclusions of this article will be made available by the authors, without undue reservation.

## Ethics statement

The studies involving humans were approved by the ethics committee of Huzhou University. The studies were conducted in accordance with the local legislation and institutional requirements. The participants provided their written informed consent to participate in this study. Written informed consent was obtained from the individual(s) for the publication of any potentially identifiable images or data included in this article.

## Author contributions

YW, PX, and HY conceived of the presented idea. YW analyzed the data and wrote up the methods and results section, and verified the analytical methods. YW and QX wrote up the first draft. PX, HY, RY, WZ, JL, and DC supervised the findings of this work. All authors contributed to the article and approved the submitted version.
